# Scientific Research Directions on the Histopathology and Immunohistochemistry of the Cutaneous Squamous Cell Carcinoma: A Scientometric Study

**DOI:** 10.3390/medicina58101449

**Published:** 2022-10-13

**Authors:** Iuliu Gabriel Cocuz, Maria Elena Cocuz, Angela Repanovici, Adrian-Horațiu Sabău, Raluca Niculescu, Andreea-Cătălina Tinca, Vlad Vunvulea, Corina Eugenia Budin, Andreea Raluca Szoke, Maria Cătălina Popelea, Raluca Moraru, Titiana Cornelia Cotoi, Ovidiu Simion Cotoi

**Affiliations:** 1 Doctoral School of Medicine and Pharmacy, University of Medicine, Pharmacy, Sciences and Technology “George Emil Palade” of Targu Mures, 540142 Targu Mures, Romania; 2 Pathology Department, Mures Clinical County Hospital, 540011 Targu Mures, Romania; 3 Pathophysiology Department, University of Medicine, Pharmacy, Sciences and Technology “George Emil Palade” of Targu Mures, 540142 Targu Mures, Romania; 4 Fundamental Prophylactic and Clinical Disciplines Department, Faculty of Medicine, Transilvania University of Brasov, 500003 Brașov, Romania; 5 Clinical Infectious Diseases Hospital of Brasov, 500174 Brasov, Romania; 6 Faculty of Product Design and Environment, Transilvania University of Brasov, 500036 Brasov, Romania; 7 Anatomy and Embryology Department, University of Medicine, Pharmacy, Sciences and Technology “George Emil Palade” of Targu Mures, 540142 Targu Mures, Romania; 8 Department of Plastic Surgery, Mures Clinical County Hospital, 540011 Targu Mures, Romania; 9 Pharmaceutical Technique Department, University of Medicine, Pharmacy, Sciences and Technology “George Emil Palade” of Targu Mures, 540142 Targu Mures, Romania; 10 Pharmacy No. 2, Mures Clinical County Hospital, 540011 Targu Mures, Romania

**Keywords:** scientometry, histopathology, squamous cell carcinoma, immunohistochemistry, dermatology

## Abstract

*Introduction:* Cutaneous squamous cell carcinoma (cSCC) is one of the most frequently occurring types of cancer in humans. Scientometric research is an innovative method for analyzing the research trends in various domains, with great implications in the field of medicine. *Materials and Methods:* We searched the Web of Science database with the following established query terms: “Squamous cell carcinoma”, “skin”, and “immunohistochemistry”. After applying the inclusion and exclusion criteria, a total of 76 articles were selected. The present study aims to analyze, based on the frequency of use of keywords with scientometric algorithms and map-based distributions, the trends of the research concerning cSCCs in 2017–2022. *Results:* A graphical representation based on 11 scientometric maps presented the division of the keywords into seven clusters, from which seven categories of research interest were defined. The clusters represent a multidisciplinary approach to the diagnosis and treatment of cSCCs, cancer diagnostics, patient outcomes, histopathological importance, management of cSCCs, role of progression, and adequate treatment of and importance of immunohistochemistry for cSCCs. The distribution of the citations shows the importance of the available research on cSCCs by analyzing the first five most-cited articles included in our study in direct concordance with the seven defined clusters. *Conclusion:* The scientometric research method reveals the interest of research in the multidisciplinary approach used to obtain the best outcomes for the patient, including a targeted investigation, as well as diagnostic and treatment options. The trends in the research reveal that histopathological diagnostics and immunohistochemistry, combined with molecular techniques, are the most important tools used to establish a personalized diagnosis, thus increasing the quality of life and life expectancy for patients with cSCCs.

## 1. Introduction

Cutaneous squamous skin carcinomas (cSCCs) are part of the non-melanocytic skin cancers (NMSCs) composed of the basal cell carcinoma (BCC), squamous cell carcinoma (cSCC), and metatypical carcinoma. The cSCC is a skin cancer based on the malignant proliferation of cells that show an increased squamous differentiation [1].

Even though, in every pathology department, the histopathological diagnosis of cSCCs is a routine procedure, it is important to mention that the successful diagnosis of these malignant tumors is also based on the immunohistochemistry panel for establishing the squamous origin of these cells.

The research concerning the diagnosis of NMSCs is continuously evolving, and, as a result of this new form of diagnostics and therapy, novel approaches are being discovered. The histopathological diagnosis of cSCCs remains the most important tool in establishing the best treatment and best outcomes for the patient [1,2].

To define the latest research trends for the histopathological and immunohistochemical diagnosis of cSCCs, we perform a scientometric study based on the scientific literature, in order to emphasize the importance as well as the necessity of the research on this kind of pathology.

## 2. Materials and Methods

**Figure 1:**
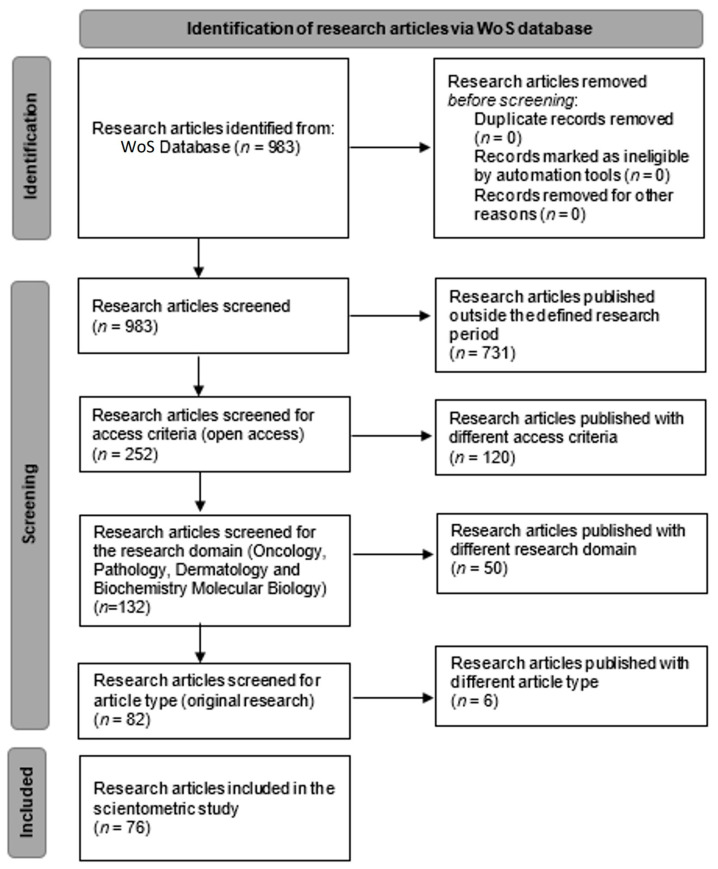
PRISMA chart of workflow—identification of research articles via WoS database.

We conducted a scientometric research study based on the available scientific literature by searching the Web Of Science (WoS) database. The WoS database was searched by defining our main research items. We established that the main research items were “squamous cell carcinoma”, “skin”, and “immunohistochemistry”. The research items were selected based on the scientometric algorithm of the research in order to organize our research target. In the WoS, the advanced search option was selected, and the query terms were set to “All fields” with the Boolean “and” value. The query for the study was: “((ALL = (Squamous cell carcinoma)) AND ALL = (skin)) AND ALL = (immunohistochemistry)”. Following the interrogation, a total of 983 articles were presented. The inclusion criteria of the articles in the present study were based on certain publication years (2017–2022), access policy (open access), research domain (oncology, pathology, dermatology, and biochemistry molecular biology), and article type (original research). The exclusion criteria were based on the values of the inclusion criteria outside the established range. The PRISMA diagram (Figure 1) shows the workflow for refining our results. After applying all the inclusion and exclusion criteria, a total of 76 articles were selected for further processing throughout our study. For assessing the impact of and to establish the research directions for squamous cell carcinomas, we used the scientometric method [3,4]. A set of instructions was defined based on the keywords from the 76 articles we assessed [5,6,7,8,9,10,11,12,13,14,15,16,17,18,19,20,21,22,23,24,25,26,27,28,29,30,31,32,33,34,35,36,37,38,39,40,41,42,43,44,45,46,47,48,49,50,51,52,53,54,55,56,57,58,59,60,61,62,63,64,65,66,67,68,69,70,71,72,73,74,75,76,77,78,79,80]. In order to obtain a valid research database of articles, the citations of the articles included in our study were analyzed with a threshold minimum of 2 citations/articles. The first five articles with the most citations were analyzed. For the graphic representation, data interpretation, and cluster formation, we used the free software VOSviewer (Version 1.6.17 – 2021 – Nees Jan van Eck and Ludo Waltman – Centre for Science and Technology Studies of Leiden University). VOSviewer is free software that can create maps of scientific data by interrogating scientific databases and creating interconnections between different parameters. The data file regarding the selected research articles from the WoS was exported as a plain text file and uploaded onto VOSviewer [81]. The scientometric analysis for our study was based on 2 attributes: the occurrence attribute and the total link strength attribute. The occurrence refers to the count of usage of the keywords in the selected articles. The total link strength indicates the total strength of the keyword links of a given research article with other research articles. Using those two attributes, we have identified the interconnections and generated the scientometric maps.

## 3. Results

The data from the selected research articles were uploaded onto VOSviewer and a series of scientometric maps were created based on the keywords obtained from the research articles; the number of occurrences of the keywords was used to represent the relationship between the keywords. A total of 400 keywords were identified from the total of 76 articles. From these keywords, a threshold minimum of two occurrences for each keyword was set. The threshold was met by 77 keywords. The 77 keywords were divided into seven clusters, based on the scientometric algorithms in terms of the occurrences of the keywords and the strength of the connection between them in the selected research articles. All the keywords included in the present study are recognized by the Medical Subject Headings 2022 (MeSH).

## 4. Cluster Classifications

**Figure 2:**
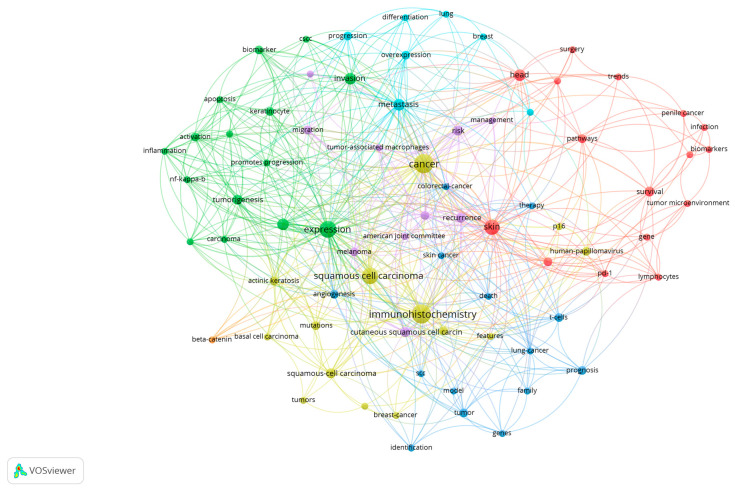
The scientometric map based on the keywords analyzed from the selected research articles *. (* Colors observed in Figure 2 refer to the defined clusters. Legend of used colors: Cluster 1—red, Cluster 2—green, Cluster 3—blue, Cluster 4—Yellow, Cluster 5—purple, Cluster 6—turquoise, Cluster 7—orange).

**Figure 3:**
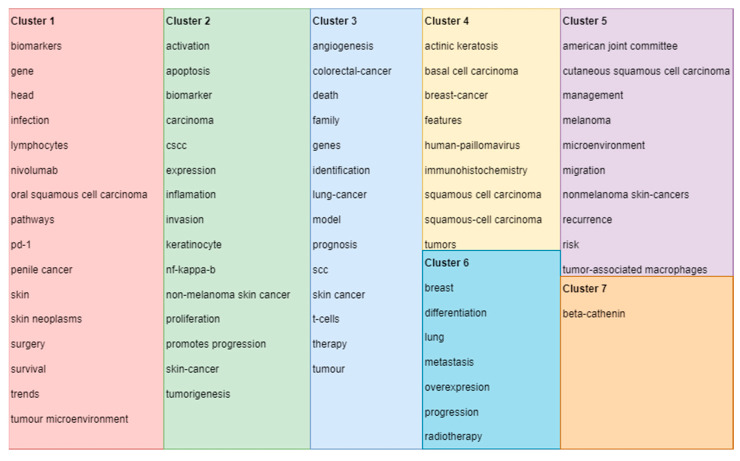
Clusters’ descriptive classifications of keywords based on the scientometric analysis *. (* Colors observed in Figure 3 refer to the defined clusters. Legend of used colors: Cluster 1—red, Cluster 2—green, Cluster 3—blue, Cluster 4—Yellow, Cluster 5—purple, Cluster 6—turquoise, Cluster 7—orange).

Cluster 1 is defined by 16 keywords obtained from the selected research articles and is shown in Figure 2 as map-based connections and in Figure 3 as descriptive classifications. Cluster 2 is defined by 15 keywords obtained from the selected research articles and is shown in Figure 2 as map-based connections and in Figure 3 as descriptive classifications. Cluster 3 is defined by 14 keywords obtained from the selected research articles and is shown in Figure 2 as map-based connections and in Figure 3 as descriptive classifications. Cluster 4 is defined by 9 keywords obtained from the selected research articles and is shown in Figure 2 as map-based connections and in Figure 3 as descriptive classifications. Cluster 5 is defined by 10 keywords obtained from the selected research articles and is shown in Figure 2 as map-based connections and in Figure 3 as descriptive classifications. Cluster 6 is defined by 7 keywords obtained from the selected research articles and is shown in Figure 2 as map-based connections and in Figure 3 as descriptive classifications. Cluster 7 is defined by 1 keyword obtained from the selected research articles and is shown in Figure 2 as a map-based connection and in Figure 3 as a descriptive classification.

## 5. Density of the Research Tendency Based on Used Keywords in the Selected Articles

**Figure 4:**
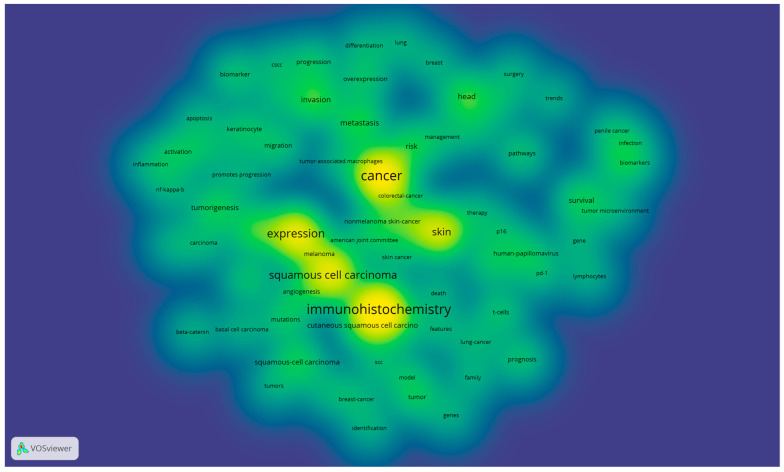
Density of the main research tendency based on used keywords in the selected articles *. (* Color legend: The intensity of the yellow–green colors shows the frequency of use of the keywords).

Figure 4 shows the density of keywords used in the selected articles based on their occurrence in the selected articles. The intensity of the yellow–green colors shows the frequency of use of the keywords.

## 6. Keyword Dynamics

The 77 examined keywords were analyzed based on the occurrence and total link strength. Table 1 shows a descriptive statistic of the two analyzed parameters.

**Figure 5:**
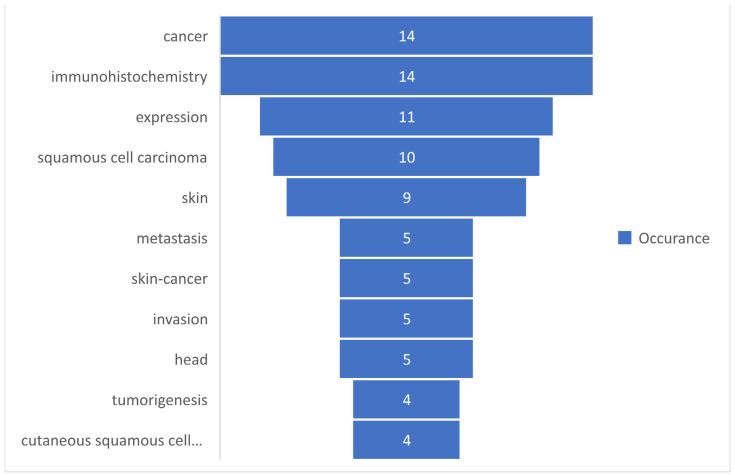
The most commonly used keywords based on occurrences.

Based on the occurrences of the keywords, a set of the first 10 most commonly used keywords was selected and presented in Figure 5.

**Figure 6:**
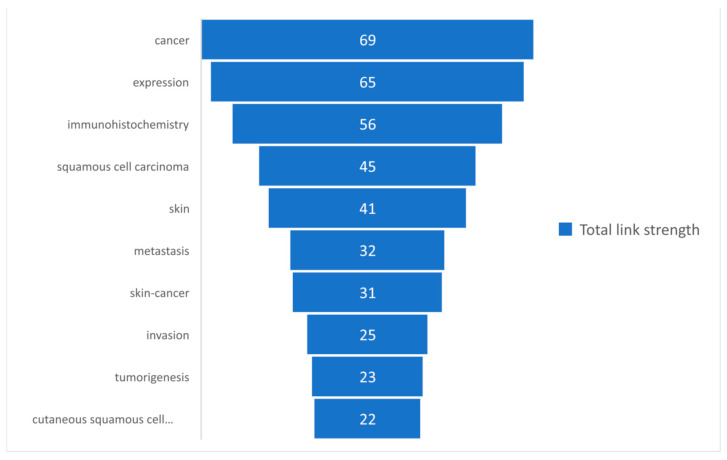
The most commonly used keywords based on the total link strength.

Based on the total link strength, a set of the first 10 most commonly used keywords was selected and presented in Figure 6.

## 7. Density of Citations in the WoS of the Selected Articles

**Figure 7:**
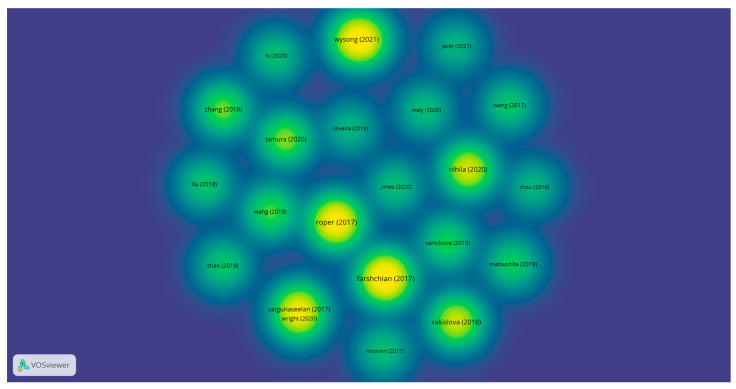
The citation density of the selected research articles from WoS *. (* Color legend: The intensity of the yellow–green colors represents the higher number of citations for the most-cited research articles out of those that were selected).

Figure 7 presents the citation density of the selected research articles from the WoS. The intensity of the yellow–green colors represents the higher number of citations for the most-cited research articles out of those that were selected.

## 8. Discussion

Squamous cell carcinomas (cSCCs) of the skin are one of the most frequently non-melanocytic skin tumors found in humans, after basal cell carcinoma [82]. Due to this fact, a series of parameters starting from clinical appearance, diagnosis, and treatment are continuously being studied to achieve the best outcomes for the patient.

The diagnosis is established mostly on the histopathological excision of the tumor, which is investigated by hematoxylin–eosin (H and E) staining and by immunohistochemistry assets. Immunohistochemistry is constantly developing, so scientific research into the panel, which is best used for the histopathological diagnosis of cSCCs, is always important.

To establish novel histopathological diagnostics, our study initially observed the main points of interest in cSCC research and combined this with immunohistochemistry. For this approach, we used the scientometric method of research. The ideology of this research method is to identify the development and transmission of scientific literature in order to analyze the most up-to-date trends in the field of interest, by using the parameters offered by scientific databases such as Web of Science.

Our study was based on defining the scientometric map based on the investigation of the keywords extracted from the WoS database by the criteria shown in the Section 2. After selecting all the parameters, the scientometric map presented in Figure 2 was generated based on the keywords that met the selected threshold. A total of seven clusters were defined by intercalating the occurrence and total link strength attributes of the analyzed keywords. The clusters defined the seven categories of research interest.

Cluster 1, composed of 16 keywords, shows the strength of the multidisciplinary approach used for cSCCs based on the following keywords: biomarkers, infection, gene, and skin. It also shows direct connections with survival, treatment options (surgery), and trends in finding new perspectives on cSCC management. Cluster 2, composed of 15 keywords, targets cancer diagnostics by establishing a direct connection between the type of cancer, in this case non-melanoma skin cancers; the category in which cSCCs are present; and presenting cell characteristics, such as apoptosis, invasion, keratinocytes, and tumor genesis. Cluster 3 presents a personal and psychological approach, in terms of the patient’s outcome, by selecting and establishing connections between different types of cancers and keywords, such as death and family. By including the keyword “therapy” in this cluster, a greater research interest in the patient outcomes and social integration can be observed. Cluster 4, from the histopathological point of view, defines the intercalation in the research with other non-melanocytic skin cancers, such as basal cell carcinomas (BCCs) of the skin, introducing the necessity of targeted immunohistochemistry for those types of cancers. Regarding cluster 5, it can be observed that the management of cSCCs remains of extremely high interest in research based on the management and recurrence of keywords and according to the American Joint Committee on Cancer. Nevertheless, cluster 6 establishes the role of progression and adequate therapy for this type of cancer in order to avoid metastasis as much as possible, which can affect the breast or even the lungs. Even though cluster 7 is formed by only one keyword, beta-catenin, it has an increased total link strength, which means that it has many connections with other keywords, and by this, it highlights an important research gateway for immunohistochemistry in the diagnosis of cSCCs. As shown in Figure 2 and Figure 3, the clusters observed on the scientometric map have a good distribution, marked by intensive connections between the keyword analyzed.

The density of the main research tendency based on the used keywords in the selected articles is presented in Figure 4, where we can observe that the interest of the scientific research concentrates on skin, cancer, expression, and immunohistochemistry, which justifies the necessity of a correct and good histopathological diagnosis based on adequate IHC panels. Nevertheless, the importance of the multidisciplinary approach to produce a good diagnosis is highlighted by the other keywords that are emerging from the most evident ones.

The keywords-review approach was based on the number of occurrences of each keyword, which was analyzed in the present study, and by the total link strength of those words. A descriptive statistic was presented to observe the tendency of using those keywords. It can be observed in Table 1 that a total of 77 keywords were analyzed, for which the maximum occurrence was 14 and the minimum was 2, with a mean rate of occurrence of 3.16. Regarding the total length strength, the minimum length strength was 3, whereas the maximum was 79. Figure 5 and Figure 6 present the 10 most frequently used keywords that had the highest occurrence and greatest total link strength. It can be observed in Figure 5 that cancer and immunohistochemistry are the most commonly used keywords, followed by expression, squamous cell carcinoma, and skin. Although our research query was defined by skin, immunohistochemistry, and squamous cell carcinoma, the keywords “cancer” and “expression” show that, in terms of the research, a great interest is shown in this type of cancer. The knowledge concerning the total link strength is changing in comparison to the occurrences, but the first four links are maintained, as shown in Figure 6. This is explained by the considerable interest of the research in this domain, based on immunohistochemistry and the expression of different parameters of squamous cell carcinomas.

In terms of citations, the density of the citations is represented in Figure 7. By analyzing the conclusions obtained for the selected research articles, our research clusters are supported by the relevant research articles. Our cluster identification and interpretation are validated by the support of the first most-cited research article. According to Satgunaseelan et al., the immunohistochemical assessment of head and neck cutaneous squamous cell carcinomas with p16 is not associated with a better prognosis. The management of the diagnosis and treatment of SCCs must be in accordance with p16 IHC expression [31]. Riihila et al. demonstrated in their study the importance of complement components C1r and C1s in cutaneous squamous cell carcinomas in terms of progression. This highlights the importance of targeted novel treatments for cSCCs [43]. Roper et al. showed that PD-L1 expression is usually observed in cSCC primary tumors and in TILs (tumor-infiltrating lymphocytes). Additionally, an expression > 5% in primary tumor cells and primary and metastatic TILs is associated with better survival rates in head and neck cSCCs. [16] Wysong et al. demonstrated that their 40-GEP genetic prognostic test, correlated with clinical and pathological findings, may improve the quality of life for the patient, presenting better patient outcomes and adequate treatment and diagnostics [35]. The results presented by Farshchian et al. in their study show the importance of AIM2 in considering the progression of cSCCs. This can lead to potential targeted therapeutic agents in primary invasive and metastatic cSCCs [32].

## 9. Conclusions

The research interest in studying squamous cell carcinomas (cSCCs) of the skin remains high, as it seeks to target new approaches in the diagnosis and treatment of this pathology. The scientometric research method revealed the necessity of a multidisciplinary approach for attaining the best outcomes for the patient. The identified scientific research directions of and interests in histopathology and immunohistochemistry in terms of diagnostics remain the most important tools used to develop a personalized diagnosis, thus increasing the quality of life and life expectancy for the patients. The limitations of the present study are represented by the fact that only the Web of Science (WoS) database was analyzed in our study.

## Figures and Tables

**Table 1:** Descriptive statistics of the occurrences and total link strength of keywords.

Occurrences		Total Link Strength	
Mean	3.168831169	Mean	15.896104
Standard Error	0.278779317	Standard Error	1.3938377
Median	2	Median	14
Mode	2	Mode	14
Standard Deviation	2.446278578	Standard Deviation	12.230876
Sample Variance	5.984278879	Sample Variance	149.59433
Kurtosis	10.69472948	Kurtosis	8.201615
Skewness	3.226291027	Skewness	2.6771373
Range	12	Range	66
Minimum	2	Minimum	3
Maximum	14	Maximum	69
Sum	244	Sum	1224
Count	77	Count	77
Confidence Level (95.0%)	0.55523713	Confidence Level (95.0%)	2.7760683

## Data Availability

All data produced here are available and can be produced upon request.
